# Rapid expansion of a left atrial myxoma caused by acute multiple internal hemorrhages: a case report and literature review

**DOI:** 10.1186/s13019-024-02495-3

**Published:** 2024-01-20

**Authors:** Takayoshi Kato, Etsuji Umeda, Natsuko Suzui, Ryo Fujii, Hiroki Ogura, Osamu Sakai, Katsuya Shimabukuro, Kiyoshi Doi

**Affiliations:** 1 Department of Cardiovascular Surgery, Gifu University Hospital, 1-1 Yanagido, Gifu City, Gifu 501-1193 Japan; 2 Department of Pathology, Gifu University Hospital, Gifu, Japan

**Keywords:** Left atrial myxoma, Internal hemorrhage, Rapid growing, SAR-CoV-2 vaccination

## Abstract

**Background:**

Left atrial myxoma is the most common benign tumor, with the growth rate remaining unknown because specific symptoms do not present until the tumor grows to a certain size. Early surgical management is performed in most cases once it is detected by physicians. Despite cardiac myxomas commonly being perceived as slow-growing tumors, rapid enlargement of myxomas has been reported.

**Case presentation:**

A 64-year-old woman was referred to our hospital with a diagnosis of a left atrial tumor. The pointed tumor changed morphologically in a few hours, and her respiratory condition, which had been normal at admission, suddenly deteriorated. Emergent surgery was performed, and the diagnosis was myxoma with multiple intratumor massive hematomas. The patient recovered uneventfully and was discharged on postoperative day 12 without any complications.

**Conclusions:**

We report an extremely rare case of left atrial myxoma rapidly expanded due to acute multiple hemorrhages within itself. Massive internal hemorrhage alters the size, shape, and fragility of the tumor. We should recognize the potential risk of internal hemorrhage that may lead to acute deterioration of the so-called “slow-growing benign” tumors, such as myxomas.

## Background

Left atrial (LA) myxomas are the most common primary heart tumors and often cause arterial embolism, cardiac flow obstruction, and sudden death. Therefore, early surgical management for LA myxomas is performed in most cases despite they are commonly perceived as slow-growing benign tumors. However, several authors have described rapid growth of LA myxomas. The causation is still unknown, but acute hemorrhage within the tumor is probably one of the important factors for rapid growth of myxomas [1–4].

Here, we report a surgically treated case of LA myxoma with acute multiple internal hemorrhages that resulted in rapid morphological collapse and symptomatic deterioration.

## Case presentation

A 64-year-old woman without any significant medical history was referred to our hospital with a diagnosis of an LA tumor.

She visited her primary clinic complaining of fatigue on light effort and stated her symptoms had been recognized after receiving her fifth vaccination for severe acute respiratory syndrome coronavirus 2 (SARS-CoV-2) four weeks earlier. Her vital signs at that time were within normal limits except her pulse rate was at a regular rhythm of 121 per minute. Echocardiography performed by the physician in the primary clinic revealed an oval tumor measuring approximately 25 × 35 mm in the LA. There was a 12-mm round hypoechoic area within the tumor. The patient had no symptoms at that time. Therefore, she drove her own car to our hospital following the instructions of the physician. Upon arrival at our hospital, she stated no symptoms at rest. Her vital signs on arrival were a blood pressure of 140/82 mmHg, a regular pulse rate of 142 per minute, and 98% oxygen saturation in room air.

**Fig. 1:**
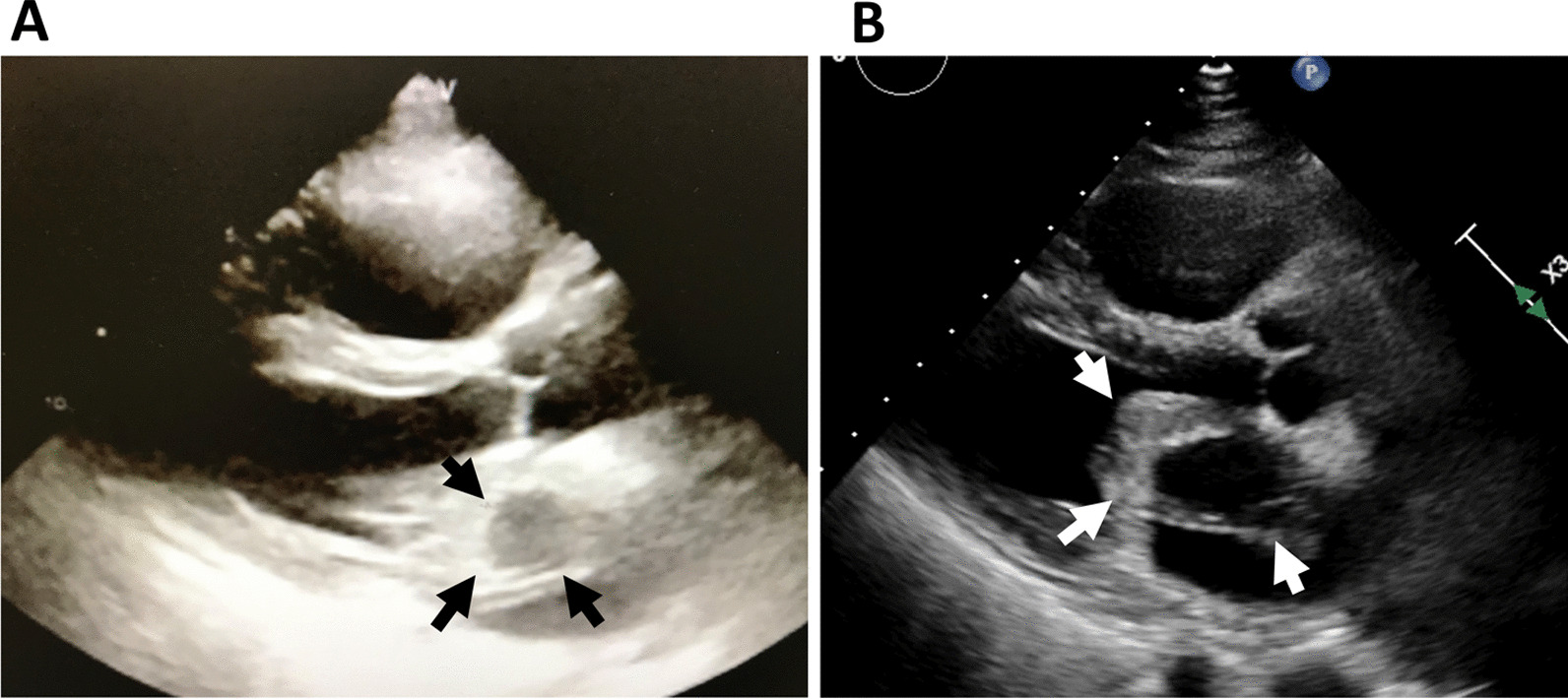
Transthoracic echocardiogram in the parasternal long-axis view. **A** An image captured by the physician in the primary clinic two hours before admission to our hospital. An oval tumor (arrows) measuring about 25 × 35 mm in the left atrium was revealed. There was a 12-mm round hypoechoic area within the tumor. **B** An image taken upon admission to our hospital. A tumor (arrows) approximately 40 × 50 mm in size included a 20 × 30-mm-sized cystic lesion within it. The tumor and its internal cystic lesion were obviously expanded compared to those in the image captured in the primary clinic two hours earlier

Transthoracic echocardiography (TTE) performed at our hospital revealed a mobile myxomatous LA tumor approximately 40 × 50 mm in size originating from the LA septum. There was a 20 × 30-mm oval cystic lesion within the tumor. The tumor and its internal cystic lesion were obviously expanded compared to those in the image captured at the primary clinic two hours earlier (Fig. 1). The mitral valve obstruction due to the expanding tumor was remarkable. Therefore, we conducted emergency surgery for tumor resection. During preparations for the surgery, the patient started complaining of dyspnea and her oxygen saturation gradually decreased to 88% under 100% of oxygen.

**Fig. 2:**
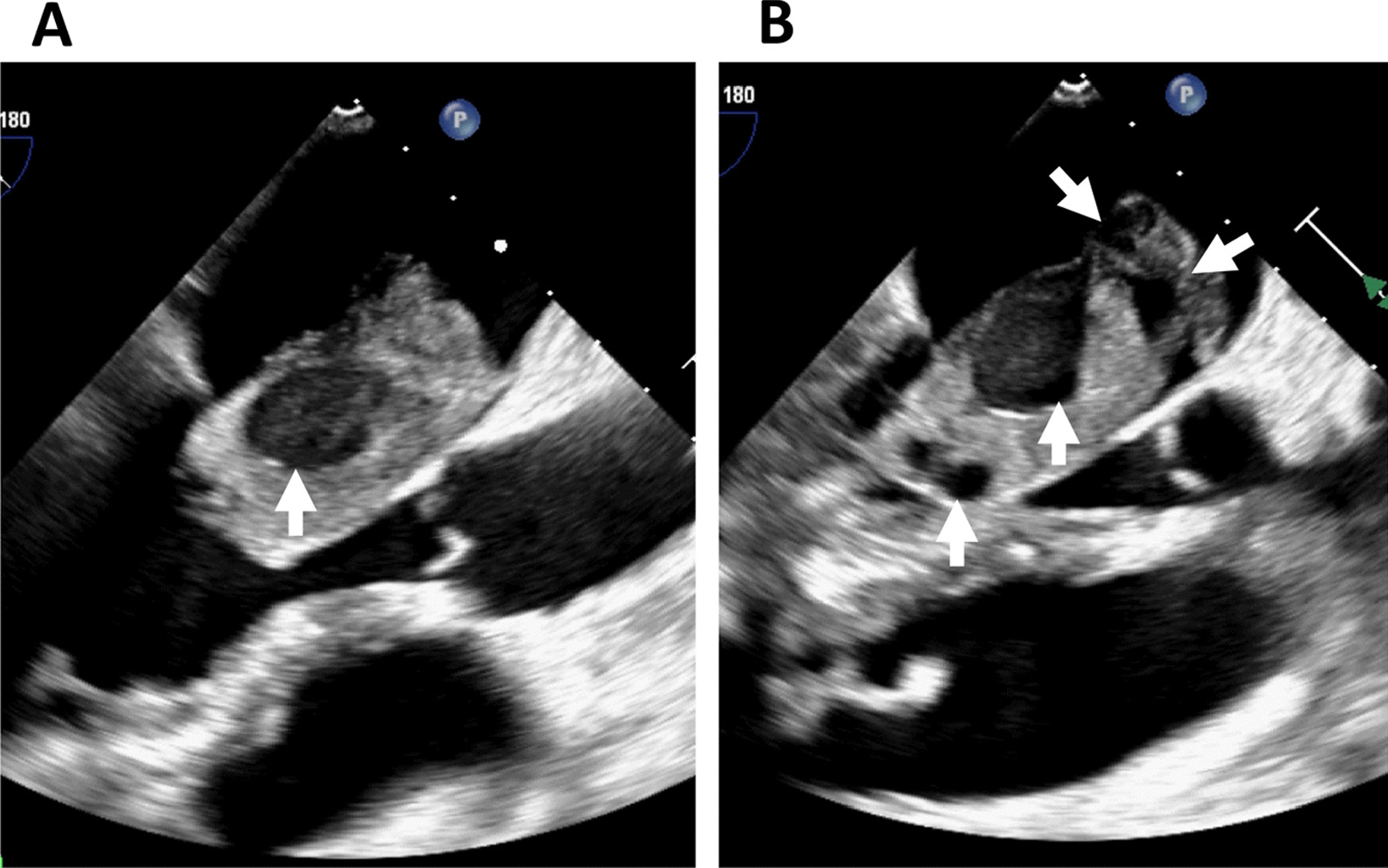
Transesophageal echocardiography images at 127 degrees. **A** At the insertion of the probe. **B** Just before establishing cardiopulmonary bypass. The tumor deformed rapidly morphologically during the operation. Flattening and lobing changes were remarkable. Small cystic formations just below the lobes were increased (arrows)

**Fig. 3:**
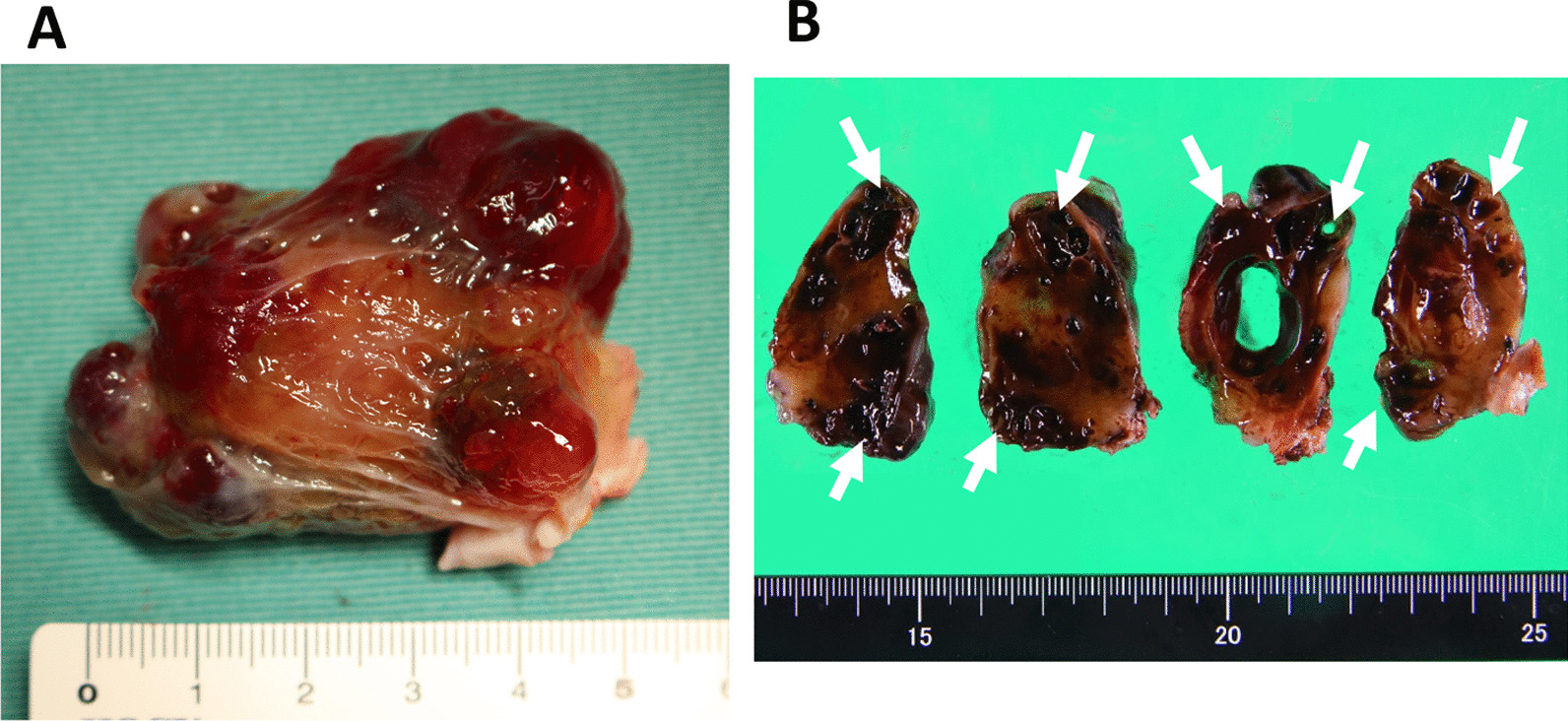
Specimen. **A** The gross appearance showing myxomatous with multiple protruding hematomas. **B** Fixed specimen showing multiple tissue defects formed by intratumor hematomas (arrows). These hemorrhages were recognized as cystic lesions on echocardiography

**Fig. 4:**
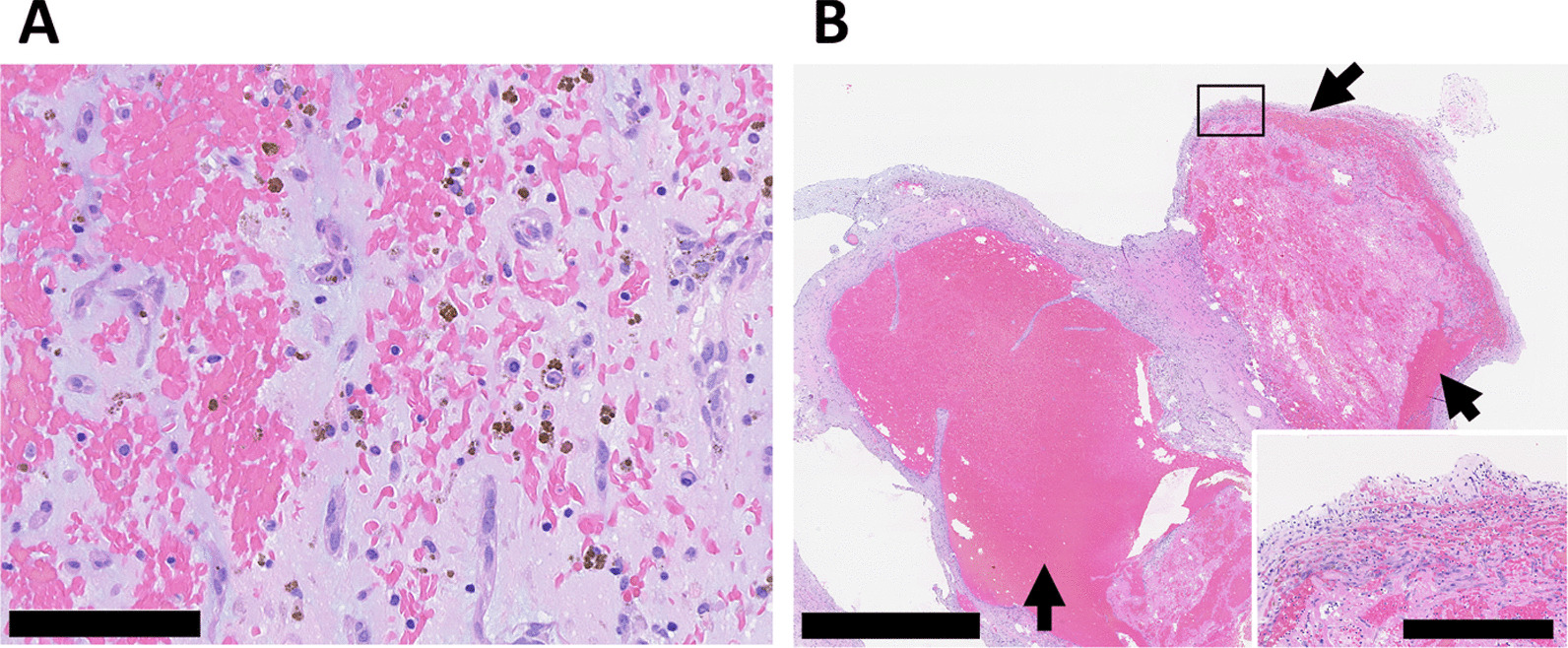
Hematoxylin and eosin-stained sections of the tumor. **A** Spindle-shaped cells and hyperchromatic nuclei segregated by abundant myxomatous stroma. Hemosiderin pigmentation and the presence of erythrocytes in the myxoid matrix indicated chronic and fresh bleeding (bar = 100 µm). **B** The myxomatous capsule right above the multiple hemorrhages was extremely thin (square) (bar = 2500 µm). The inset shows a thin capsule under high magnification (bar = 500 µm). Arrows indicate hemorrhages

In the operating room, transesophageal echocardiography (TEE) disclosed that the tumor had been morphologically changing rapidly over time. Longitudinal expansion and lobing changes were remarkable (Fig. 2). Under cardiopulmonary bypass and cardioplegic arrest, a transseptal supra-atrial incision was made. The tumor pedicle was attached to the inter-atrial septum and the free LA wall. Resection of the tumor with the surrounding atrial wall and reconstruction of the defects using a bovine patch were performed. The myxomatous mass with multiple protruding hematomas measured 50 × 38 mm. Multiple myxomatous tissue defects were formed due to fresh intratumor bleeding (Fig. 3). Microscopic findings showed spindle-shaped cells and hyperchromatic nuclei segregated by abundant myxomatous stroma. Hemosiderin pigmentation and the presence of erythrocytes in the myxoid matrix indicated chronic and fresh bleeding. The myxomatous capsule right above the site of multiple hemorrhages was extremely thin (Fig. 4). The postoperative recovery was uneventful, and the patient was discharged on postoperative day 12 without any complications.

## Discussion and conclusions

In general, cardiac myxomas are recognized as slow-growing benign tumors. However, because of their potentially life-threatening manifestations, such as arterial embolism and cardiac flow obstruction, early surgical management has become a common practice in recent years.

The growth rate of LA myxomas was first described in 1987 [5, 6] and has been reported in several studies since then. After searching the English literature on the study of the growth rate of primary LA myxoma, we found 16 reports mentioning the growth rate [5–20] (Table 1). The median value and range of growth rate were 3.5 mm/month and 0–28.3 mm/month, respectively. Walpot et al. [14] conducted a literature review and disclosed a “rapid-growth rate” of LA myxoma averaging 4.9 mm/month. Regarding its growth direction, Karlof et al. [21] assumed that myxomas grow in a linear fashion. However, in reality, growth may be exponential such that the estimated growth rate would vary depending on when the diagnosis is made. Iga et al. [11] reported a case in which the horizontal and longitudinal distances of the tumor increased, while the size of the base remained unchanged.

**Table 1:** Reported growth rate of left atrial myxoma

Author	Year	Age (y)/sex	Size first echo (mm)	Size last echo (mm)	Interval (months)	Growth rate (mm/months)
Marinissen et al. [5]	1987	65 /male	Absent	60 × 40	18	3.3
Roudaut et al. [6]	1987	45/male	Absent	60 × 40	7	8.5
Ahern et al. [7]	1989	76/male	Absent	25 × 40	17	2.3
Pochi et al. [8]	1991	62/female	Absent	75 × 50 × 35	17	4.4
Rey et al. [9]	1993	74/female	Absent	45 × 29 × 39	27	1.7
Lane et al. [10]	1994	71/male	29 × 21	28 × 21	28	0
Iga et al. [11]	1997	57/male	15 × 13	38 × 36	18	2.1
Kay and Chow [12]	2002	71/male	40 × 46	40 × 47	180	0
Ullah and McGovern [13]	2005	89/male	NA	NA	79	0.04
Walpot et al. [14]	2010	65/female	Absent	44 × 40	12	3.7
Vazir and Douthwaite [15]	2011	62/female	Absent	26.7 × 10	12	2.2
Alvarez et al. [16]	2013	60/male	10 × 10	20 × 20	3	6.6
Kim and Kim [17]	2019	75/female	Absent	38 × 27	3	12.6
Strecker et al. [18]	2019	65/male	Absent	12 × 17 × 17	12	1.4
Longhitano et al. [19]	2021	71/female	10 × 6	36 × 29	8	28.3
Goodwin et al. [20]	2021	32/female	NA	NA	4.5	7.7

*NA* not applicable

Rubio Alvarez et al. [16], based on the case they encountered, noted that the initial period of tumor growth can be quite rapid. In contrast, Lane et al. [10] stated that LA myxoma may exhibit a quiescent phase or at least a heterogeneous growth rate. Though reporting bias must be considered, these presentations might support the speculation that myxomas grow faster than they are considered to.

In our case, although there were no data concerning the growth rate of the myxoma, it is worth mentioning that the tumor demonstrated acute multiple hemorrhages within itself, which were recognizable as multiple cystic lesions during echocardiography. The pathological study revealed massive fresh central hemorrhage and lobular subcapsular bleeding accompanying the old hemorrhage. Roskell and Biddolph [2] have documented that clinical cases in which myxomas have grown rapidly are probably due to changes in the intercellular matrix rather than cellular proliferation. In our case, multiple intratumor hemorrhages pushed the parenchyma outward, and the outer layer of the myxoma was extremely thin. TEE proved the rapid longitudinal expansion and lobing changes over time.

The massive internal bleeding of myxomas, like in our case, is an extremely rare manifestation reported in English literature [1, 3, 4]. These reports describe rapid clinical deterioration and a cystic lesion within the myxoma. However, multiple cystic lesions due to intra-myxoma hemorrhage have not been reported previously. In our review of case reports mentioning obvious growth rates, we were unable to find reports on myxomas with cystic lesions. In other words, myxomas with massive bleeding might show extremely quick growth. We believe that this report is the first to present a highly uncommon occurrence of acute multiple internal hematomas, accompanied by a notably accelerated morphological transformation and collapse in a myxoma, induced by acute extensive intratumoral bleeding.

The pathological examination of the tumor showed abnormally dilated vessels around the tumor stalk, and their disruption was thought to be the cause of internal hemorrhages. However, the cause of the bleeding within the myxoma is unknown. The patient denied any history of chest trauma or bleeding tendency. Her laboratory test results did not reveal any abnormality in coagulation and fibrinolytic system. Recently, rare cases of acute pituitary tumor hemorrhage after SARS-CoV-2 vaccination have been reported [22, 23]]. Regarding the current case, four weeks before emergent surgery, the patient received the fifth SARS-CoV-2 vaccination and noticed that she felt abnormally fatigued on light exertion one week after the inoculation. Although we cannot confirm the correlation between intratumor bleeding and vaccination, this case may also call attention to possible intratumor hemorrhage after SARS-CoV-2 vaccination.

In conclusion**,** we report an extremely rare case of LA myxoma rapidly expanded due to acute multiple internal hemorrhages. Massive internal hemorrhage changes tumor size, shape, and fragility. We should recognize the potential risk of internal hemorrhage that may lead to acute deterioration of the so-called “slow-growing benign” tumors, such as myxoma.

## Data Availability

Data sharing is not applicable to this article as no datasets were generated or analyzed.

## Abbreviations

LA: Left atrial
SARS-CoV-2: Severe acute respiratory syndrome coronavirus 2
TTE: Transthoracic echocardiography
TEE: Transesophageal echocardiography
